# Longitudinal Analysis of the Effect of Repeated Transarterial Chemoembolization for Liver Cancer on Portal Venous Pressure

**DOI:** 10.3389/fonc.2021.639235

**Published:** 2021-11-05

**Authors:** Constantine Frangakis, Jae Ho Sohn, Ahmet Bas, Julius Chapiro, Ruediger E. Schernthaner, MingDe Lin, James P. Hamilton, Timothy M. Pawlik, Kelvin Hong, Rafael Duran

**Affiliations:** ^1^ Department of Biostatistics, The Johns Hopkins Bloomberg School of Public Health, Baltimore, MD, United States; ^2^ Russell H. Morgan Department of Radiology and Radiological Science, Division of Vascular and Interventional Radiology, The Johns Hopkins Hospital, Baltimore, MD, United States; ^3^ Department of Radiology, İstanbul University Cerrahpaşa Medical School, İstanbul, Turkey; ^4^ Department of Radiology and Biomedical Imaging, Yale University School of Medicine, New Haven, CT, United States; ^5^ Division of Gastroenterology and Hepatology, Department of Medicine, The Johns Hopkins School of Medicine, Baltimore, MD, United States; ^6^ Department of Surgery, The Ohio State University, Wexner Medical Center, Columbus, OH, United States; ^7^ Department of Radiology and Interventional Radiology, Lausanne University Hospital and University of Lausanne, Lausanne, Switzerland

**Keywords:** portal hypertension, longitudinal data analysis, transarterial chemoembolization, TACE, hepatocellular carcinoma, HCC, variceal bleeding

## Abstract

**Objectives:**

Investigate long-term effects of repeated transarterial chemoembolization (TACE) on portal venous pressure (PVP) using non-invasive surrogate markers of portal hypertension.

**Methods:**

Retrospective, Institutional Review Board-approved study. 99 patients [hepatocellular carcinoma (HCC) group (n=57); liver metastasis group (n=42)] who underwent 279TACEs and had longitudinal pre-/post-therapy contrast-enhanced-MRI (n=388) and complete blood work were included. Outcomes of interest were platelet count (PC), spleen volume, ascites and portosystemic collaterals. Variables included TACE type/number, tumor type, microcatheter location, Child-Pugh, baseline tumor burden (tumor number/total/largest size), vessel invasion, alpha-fetoprotein, Eastern Cooperative Oncology Group (ECOG) performance status, and Model for End-Stage Liver Disease (MELD) score. Generalized Estimating Equations assessed the associations between TACE and outcomes. Power analysis determined the sample size was sufficient.

**Results:**

No significant change in PC over time was observed in either groups, regardless of liver function (*P*>0.05). Baseline spleen volume was 226 cm^3^ for metastatic group, and was larger by 204 cm^3^ for HCC group (*P*<0.001). Spleen volume increased by 20 cm^3^ (95%CI: 8-32; *P*=0.001) for both groups after 1^st^TACE and by 16cm^3^/TACE (*P*=0.099) over the full follow-up (up to 9TACEs). Spleen volume also tended to increase by 23cm^3^ (95%CI: -1–48; *P*=0.064) with higher tumor burden. Odds of developing moderate/severe ascites for metastatic patients was decreased by 0.5 (95%CI: 0.3–0.9; *P*=0.014), regardless of the Child-Pugh, and increased by 1.5 (95%CI: 1.2–1.9; *P*<0.001) among HCC patients with unstable Child-Pugh, whereas no change was noted with stable Child-Pugh. HCC patients with unstable Child-Pugh demonstrated a significant increase in portosystemic collaterals number over time (*P*=0.008). PVP-related complications such as variceal bleeding post-TACE were low (0.4%).

**Conclusion:**

Repeated TACEs did seem to have an impact on PVP. However, the increase in PVP had marginal effects with low portal hypertension-related complications.

## Introduction

Portal hypertension is detrimental and may be complicated by the development of ascites, splenomegaly with thrombocytopenia, gastroesophageal varices formation and rupture, portal hypertensive gastropathy, hepatic encephalopathy and hepato-renal or pulmonary syndromes (1).

Direct measurement of portal venous pressure (PVP) by puncturing the portal vein is the most accurate assessment of portal hypertension. This procedure is, however, invasive and only performed in specific indications. Hepatic venous pressure gradient (HVPG) measurement is the preferred method for the indirect assessment of portal hypertension (1–3). However, the routine use of HVPG is not recommended because of its invasiveness, cost, and need for technical expertise, and development of noninvasive surrogate markers of portal hypertension is encouraged (4).

Transarterial chemoembolization (TACE) is the standard of care for intermediate-stage patients with hepatocellular carcinoma (HCC) and is increasingly used to treat metastatic disease to the liver (5, 6). Theoretically, TACE primarily targets tumor tissue. However, inevitably, part of the administered treatment reaches non-tumorous hepatic parenchyma – known as non-targeted drug delivery. Nontumoral liver tissue is also dependent upon the hepatic artery for oxygenated blood, so embolization-induced ischemia can damage liver tissue and potentially increase PVP over time (7, 8).

Scarce and contradictory data exists about portal hemodynamic changes post-TACE. Invasive measurements using percutaneous transhepatic (9) or transvenous (HVPG) (10) routes performed immediately before and after TACE have not demonstrated any significant change post-procedure in HCC patients. HVPG measured within 9 days and 2 months after TACE did not significantly change from measurements prior therapy, but did increase at 6 months (11). TACE resulted in a significant increase in esophageal variceal pressure in about half of HCC patients with most (89%) showing an increase in portal blood flow (PVBF) on ultrasound 3 days post-procedure (12). PVBF was noted to increase after TACE, reaching a peak at 1 week and returning to pre-procedural values after 3-4 weeks (9, 13) or remaining elevated for at least 2 weeks post-embolization (14). In contrast, no change in PVBF post-TACE was reported (15).

We aimed to investigate long-term effects of repeated TACE on PVP within the context of a longitudinal analysis using non-invasive surrogate markers of portal hypertension.

## Materials and Methods

This retrospective single-institution study was compliant with the Health Insurance Portability and Accountability Act and approved by the Institutional Review Board. Informed consent was waived.

### Study Design and Groups

A prospectively collected database of patients who underwent TACE was reviewed and 160 consecutive patients were identified. As liver cirrhosis directly impacts PVP, two study groups were evaluated: HCC patients with cirrhosis and patients with metastatic liver disease without cirrhosis.

Inclusion criteria were: HCC diagnosed by biopsy and/or cross-sectional imaging (5), biopsy-proven liver metastasis, contrast-enhanced MRI and complete blood work before/3-6 weeks after each TACE. Exclusion criteria: patients with metastatic liver disease and cirrhosis confirmed by liver biopsy or MR findings suggesting cirrhotic liver (n=1) (16), absence of baseline or follow-up MRI (n=3) or compete blood work (n=23), image artifacts (n=2), incomplete spleen coverage (n=19), splenectomy (n=8), spleen infarction (n=1), splenic vein thrombosis (n=1), diffuse-type HCC (n=3) (9). The final study population included 99 patients.

### Non-Invasive Surrogate Markers of Portal Hypertension

Noninvasive techniques for the assessment of portal hypertension have been validated (4). Established surrogate markers for portal hypertension include platelet count, spleen volume, ascites or portosystemic collaterals (4, 17–22). These variables were investigated longitudinally before and after each TACE. Other longitudinally investigated variables were: portal vein thrombosis (PVT), hepatic vein and inferior vena cava (IVC) thrombosis, number of TACEs, TACE-type [conventional (cTACE) *vs*. drug-eluting beads (DEB-TACE)], microcatheter location [proximal (lobar) *vs*. distal (segmental/subsegmental)] and delivered payload (doxorubicin dose; volume of Lipiodol and bland beads, and DEBs), baseline tumor burden (tumors number, total tumor and largest tumor size), Eastern Cooperative Oncology Group (ECOG) performance status, Model for End-Stage Liver Disease (MELD) score, and Alpha-fetoprotein (AFP). As portal hypertension is correlated with liver function, Child-Pugh class/score were calculated at baseline and after each TACE to ensure that observed signs of increased PVP were not related to a decrease in liver function over time (23–25).

Medical records were thoroughly reviewed and the advent of any portal hypertension-related complication (such as upper/lower gastrointestinal bleeding, encephalopathy, hepato-renal/pulmonary syndromes, worsening or severe ascites needing paracentesis) was recorded.

TACE Protocol (**Supplementary Material 1**)

MRI Protocol (**Supplementary Material 2**)

### MR Images Analysis

MR images were evaluated by two expert radiologists (A.B. and R.D., with 13 and 10 years of experience). Readers were blinded to any clinical information. Quantitative semiautomatic tissue segmentation software (Medisys, Philips Research, Suresnes, France) was used to obtain the spleen volume (portal venous phase). This method is accurate and highly reproducible (26). Spleen volumes were measured independently and results were averaged. Ascites was graded on a three-point scale (mild-moderate-severe). Portosystemic collaterals were categorized as paraumbilical, anterior abdominal wall, gastric (left/short gastric veins), para-/esophageal, retroperitoneal (including spleno-/gastro-renal, paravertebral) and mesenteric varices (27). Varices were defined as tubular, tortuous, serpiginous, dilated vascular structures with > 3 mm in diameter except for para- and esophageal varices for which a diameter of > 2 mm was used (28). PVT was classified on a five-point scale (main-lobar-sectorial-segmental-subsegmental). Hepatic vein thrombosis with potential IVC and right atrium extension was noted. Measurement of portal vein diameter has low sensitivity and poorly correlates with the degree of portal hypertension, and was not performed (29, 30). As interobserver variability has been reported in the assessment of variables (31), presence and categorization of ascites, portosystemic collaterals and venous thrombosis were determined in consensus.

### Statistical Analysis

Data were summarized using descriptive statistics (count and frequency for categorical variables and mean and range for continuous variables). Each of four outcomes of interest (platelet count, spleen volume, ascites and portosystemic collaterals) was visualized, explored, and analyzed, in relation to key aspects of the dataset as well as to understand the general trend of relationship between TACEs and outcomes.

For visualization, spaghetti plots were created to observe each outcome’s trajectories over time, within subgroups based on tumor type, Child-Pugh stability, or number of visits.

For both exploratory and final analysis, and because of the longitudinal nature of the study and the non-uniform numbers of repeated measurements, we used Generalized Estimating Equations (GEE) (32), where the visit number (=TACE number) in which a measurement takes place was used as a covariate. The exploratory analysis considered several factors as possible predictors of the outcomes, based on the clinical understanding of the disease process as well as factors of significant interest to clinicians. The initially chosen predictors for an outcome at a visit included the number of previous visits, tumor type (HCC *vs*. metastasis), whether patient’s Child-Pugh class remained stable throughout the course of the treatment, baseline tumor burden, TACE type (cTACE *vs*. DEB-TACE *vs*. combination), microcatheter location, whether there was vessel invasion at that visit, AFP, MELD, and ECOG performance status. Also, based on the pattern observed in the visualization, a quadratic term of “visit” was included in the initial factors. From those initial predictors, we use a combination of clinical knowledge and sensitivity analysis, to select the final predictors. In particular, for each outcome, we used Akaike Information Criterion (AIC) to select first, which interactions among number of visits, tumor type, total tumor size, and Child-Pugh stability are important; and second, which “main effects” the remaining predictors were important. For the final analyses, we examined the degree to which each outcome was related to the final predictors using the respective GEE. The identity link was used for platelet count, spleen volume, and portosystemic collaterals, and the logit link was used for the probability of presence of ascites. Calendar time was temporarily added as a predictor to explore the correlation and interchangeability with the number of visits. A power analysis determined the sample size was sufficient to detect with high probability the substantial effects we observed in the study for the relation between additional visits and the outcomes (power>94%). Statistical analysis was performed with R (R Core Team, 2014).

## Results

### Patient Data

Baseline patient and TACEs characteristics are summarized in **Table 1**. 84 patients (84.9%) underwent up to 4 procedures and 15 patients up to 9 procedures (5 to 9 TACEs). A mean of 2.8 ± 1.7 TACEs [range, 1-9] were performed per patient. 207 (74.2%) TACEs were performed on the right liver lobe, 53 (19.0%) on the left, and 19 (6.8%) on both lobes. Metastatic group patients had significantly higher baseline tumor burden when compared to the HCC group (number of tumors and total tumor size, both *P*<0.001).

**Table 1:** Baseline Patient and TACEs Characteristics.

Characteristic	HCC group	Metastatic group
	Value (%)	Value (%)
No. of Patients	57	42
Sex		
Female	14 (24.6)	25 (59.5)
Male	43 (75.4)	17 (40.5)
Age*		
All patients	62 (12.1) [range, 19-85]	57 (11.5) [range, 19-80]
Female	61 (9.3) [range, 46-80]	55 (12.4) [range, 19-74]
Male	62 (13.0) [range, 19-85]	59 (9.7) [range, 42-80]
Ethnicity		
White	33 (57.9)	40 (95.2)
African American	10 (17.5)	2 (4.8)
Hispanic	2 (3.5)	0 (0)
Other	12 (21.1)	0 (0)
ECOG Performance Status		
Grade 0	26 (45.6)	25 (59.5)
Grade 1	24 (42.1)	17 (40.5)
Grade 2	7 (12.3)	0 (0)
Etiology		
HCV	15 (26.3)	
HBV	14 (24.6)	
Cryptogenic	11 (19.3)	
HVC+EtOH	8 (14.0)	
EtOH	5 (8.8)	
NASH	3 (5.3)	
Hemochromatosis	1 (1.8)	
Neuroendocrine tumor		26 (61.8)
Breast cancer		7 (16.7)
Sarcoma		7 (16.7)
Pancreatic cancer		1 (2.4)
Colorectal cancer		1 (2.4)
Child-Pugh Class		
A	42 (73.7)	40 (95.2)
B	14 (24.6)	2 (4.8)
C	1 (1.7)	0 (0)
Child-Pugh Score*	6 (1.2) [range, 5-10]	5 (0.55) [range, 5-7]
MELD score*	6 (3.5) [range, 0-16]	NA
Biopsy Proven HCC/Metastasis	29 (50.9)	42 (100)
Alpha-fetoprotein*	6477 (39820) [range, 1-300308]	NA
Number of Tumors*	2.7 (3.9) [range, 1-25]	35.6 (42.4) [range, 2-224]
Largest Tumor [cm]*	8.2 (4.2) [range, 2.7-20.2]	7.6 (3.9) [range, 2.1-18.4]
Total Tumor Size [cm]*^‡^	13.7 (13.7) [range, 2.7-75.4]	88.8 (123.8) [range, 3.6-696.8]
Portal Vein Thrombosis	3 (5.3)	5 (8.4)
Total TACE procedures	170	109
cTACE	145 (85.3)	87 (79.8)
DEB-TACE	25 (14.7)	22 (20.2)
Treatment Modality		
cTACE only	43 (75.4)	30 (71.4)
DEB-TACE only	5 (8.8)	7 (16.7)
Crossover	9 (15.8)	5 (11.9)
Position of microcatheter (all TACEs)		
Proper hepatic artery	3 (5.3)	2 (4.8)
Right or left hepatic artery	40 (70.2)	35 (83.3)
Other (sectorial, sub-segmental or segmental)	14 (24.5)	5 (11.9)
Child-Pugh Class evolution (baseline→end of follow-up)		
A→A	26 (45.6)	29 (69)
A→B	14 (24.6)	11 (26.2)
A→C	2 (3.5)	0
B→B	10 (17.5)	2 (4.8)
B→C	4 (7)	0
C→C	1 (1.8)	0

Except where indicated, data represents numbers of patients with percentages in parentheses. *Data represented as mean (standard deviation) and range. ^‡^Obtained by adding all liver lesions. NA, not applicable.

388 MRI were reviewed; mean MRI per patient was 3.8 ± 1.7 (range, 2-10). Mean time between pre-treatment MRI/labs to the first TACE was 2.4 ± 2.0 weeks (range, 0-7.1)/2.5 ± 2.5 weeks (range, 0-9.9), respectively. Mean time from the first TACE to post-treatment MRI/labs was 3.8 ± 1.4 weeks (range, 0.9-10)/4.1 ± 1.8 weeks (range, 0-11.1), respectively. Mean time from each subsequent TACE to post-treatment MRI/labs was 4.2 ± 1.7 weeks (range, 0.9-11)/4.5 ± 2.0 weeks (range, 0.6-12.3), respectively. Mean follow-up period for the study population was 23.5 ± 25.5 months (range, 0.5-134.6).

### Longitudinal Analysis of Platelet Count

Mean baseline platelet count was 239x10^9^/L (95%CI:215-263) for the metastatic group, and was lower by 58x10^9^/L units for the HCC group (*P*=0.007) (**Table 2A**). Child-Pugh stability was judged as not predictive by the model. Between baseline and the first TACE, platelet count changed significantly by 72x10^9^/L (95%CI:26-117;*P*=0.002) for the metastatic group, but not for the HCC group. However, over the full follow-up of up to nine TACEs, platelet count for the metastatic group dropped back to baseline levels and there was no difference from baseline. Similarly, no significant change in platelet count over time was observed in the HCC group (*P*=0.336) (**Table 2A**).

**Table 2:** Longitudinal analysis of platelets and spleen volume.

A	Average (+ change) of Platelet Values					
	visits 0-1 used			all visits used		
	Estimate	95%CI	*P* (for 0)	Estimate	95%CI	*P* (for 0)
for MET group at visit 0, with unstable Child-Pugh	239	215:263				
+ HCC group	-58	-101:-16	0.007			
+ stable Child-Pugh	–	–	–			
+ additional visit						
If MET, any Child-Pugh	72	26:117	0.002	0	-20:19	0.978
If HCC, any Child-Pugh	0	-18:19	0.978	-4	-13:4	0.336
+ 1 sd of Total tumor size at baseline	–	–	–	–	–	–
+ vascular invasion	–	–	–	–	–	–
						
**B**	**Average (+ change) of Spleen Volume**					
	**visits 0-1 used**			**all visits used**		
	**Estimate**	**95%CI**	***P* (for 0)**	**Estimate**	**95%CI**	***P* (for 0)**
for MET group at visit 0, with unstable Child-Pugh	226	192:261				
+ HCC group	204	125:282	<0.001			
+ stable Child-Pugh	–	–	–			
+ additional visit	20	8:32	0.001	16	-3:36	0.099
If MET, any Child-Pugh						
If HCC, any Child-Pugh	(same as MET)			(same as MET)		
+ 1 sd of Total tumor size at baseline	23	-1:48	0.064	–	–	–
+ vascular invasion	-23	-118:72	0.635	–	–	–

Child-Pugh Stability, Visit: Child-Pugh Stability, Largest Tumor Size at Baseline, Number of Tumors at Baseline, TACE type, Microcatheter Location, AFP, MELD and ECOG dropped during model selection per AIC; dashed lines indicate that the corresponding variable was selected out by the model selection process as unimportant. Visit = TACE procedure.

Baseline largest tumor size and number of tumors, TACE type/selectivity, AFP/MELD values, and ECOG did not have an effect on platelet count as these factors were dropped during model selection per AIC.

**Figure 1:**
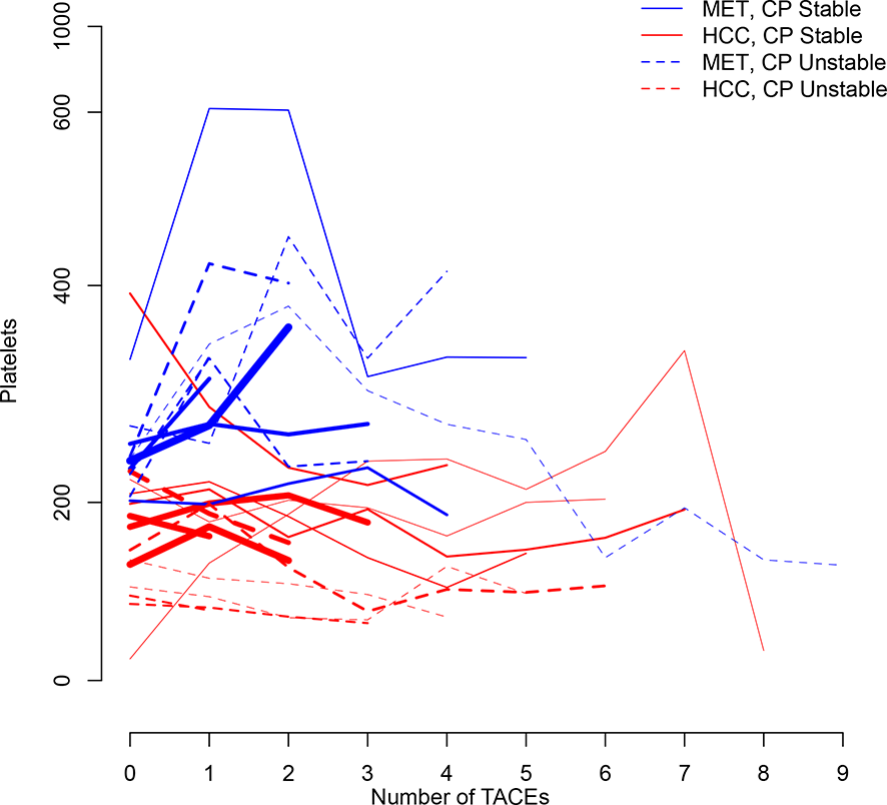
Trajectory plot illustrating longitudinal changes over time in platelet count according to the number of TACEs and stratified by Child-Pugh (CP) stability (line = stable, dotted line = unstable) and metastatic (MET)/HCC groups. Line thickness is proportional to patient number (the thicker the line the more patients).

**Figure 1** illustrates longitudinal changes over time in platelet count.

### Longitudinal Analysis of Spleen Volume

Baseline mean spleen volume was 226cm^3^ (95%CI:192–261) for the metastatic group, and was larger by 204cm^3^ for the HCC group (*P*<0.001) (**Table 2B**). Child-Pugh stability was not predictive by the model. Between baseline and the first TACE, mean spleen volume changed significantly by 20cm^3^ (95%CI:8-32;*P*=0.001) for all groups (**Table 2B**). Over the full follow-up (up to 9 TACEs), mean spleen volume was estimated to have increased by 16cm^3^/TACE (again, regardless of Child-Pugh status or metastatic *vs*. HCC group) (*P*=0.099). Mean spleen volume also marginally increased by 23cm^3^ (95%CI:-1-48;*P*=0.064) for every increase in total tumor size by one standard deviation (88.6cm) (**Table 2B**).

Baseline largest tumor size and number of tumors, TACE type/selectivity, AFP/MELD values, and ECOG did not have statistically significant effect on spleen volume.

**Figure 2:**
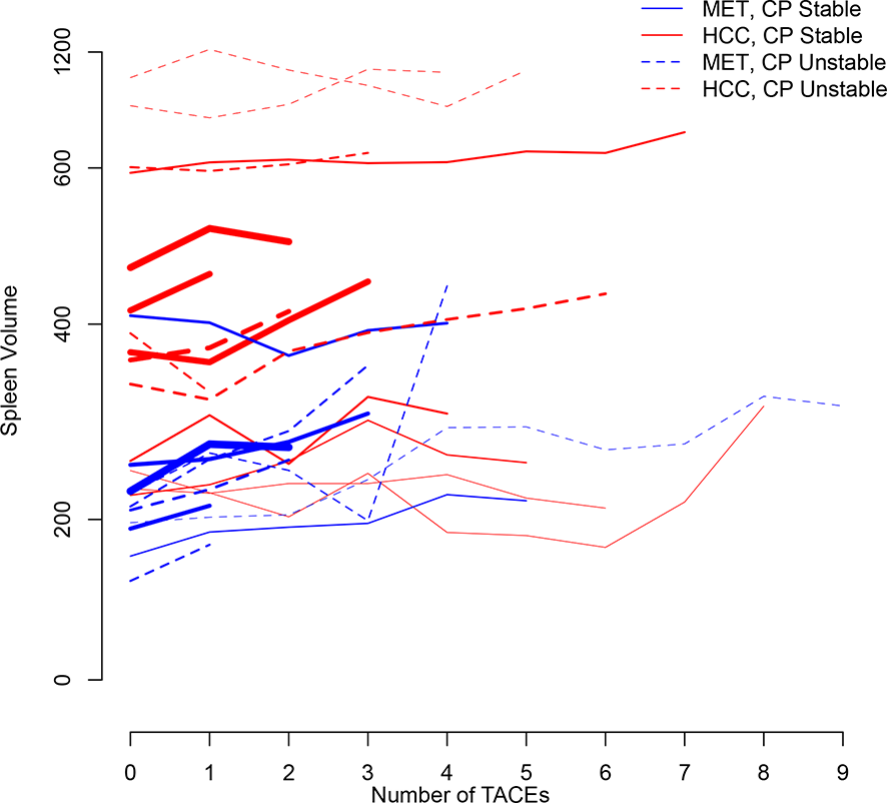
Trajectory plot illustrating longitudinal changes over time in spleen volume according to the number of TACEs and stratified by Child-Pugh (CP) stability (line = stable, dotted line = unstable) and metastatic (MET)/HCC groups, respectively. Line thickness is proportional to patient number (the thicker the line the more patients).

**Figure 2** illustrates longitudinal changes over time in spleen volume.

### Longitudinal Analysis of Ascites

No metastatic patient had baseline moderate/severe ascites. HCC patients did not develop moderate/severe ascites after the first TACE (**Table 3A**). Child-Pugh status was not predictive of developing moderate/severe ascites after the first TACE for both groups. Over the full follow-up, the odds of developing moderate/severe ascites for the metastatic group was decreased by 0.5 (95%CI:0.3–0.9;*P*=0.014), regardless of the Child-Pugh status (**Table 3A**). No significant change in the odds of developing moderate/severe ascites over time was observed in HCC patients with stable liver function. However, the odds of developing moderate ascites/severe significantly increased over time by 1.5 (95%CI:1.2–1.9;*P*<0.001) in HCC patients with unstable Child-Pugh (**Table 3A**).

**Table 3:** Longitudinal analysis of ascites and portosystemic collaterals formation.

A	Odds (+ Odds Ratios) of Having Moderate/Severe Ascites					
	visits 0-1 used			all visits used		
	Estimate	95%CI	*P* (for 1)	Estimate	95%CI	*P* (for 1)
for MET group at visit 0, with unstable Child-Pugh	0.0	0.0:0.1	–			
+ HCC group	–	–	–			
+ stable Child-Pugh	0.9	0.1:5.8	0.931			
+ additional visit						
If MET, any Child-Pugh	2.5	0.6:9.7	0.198	0.5	0.3:0.9	0.014
If HCC, unstable Child-Pugh	1.7	0.1:20.8	0.685	1.5	1.2:1.9	<0.001
If HCC, stable Child-Pugh	1.8	0.2:13.8	0.560	1.0	0.8:1.4	0.756
+ ECOG	–	–	–	–	–	–
						
**B**	**Average (+ change) in Number of Porto-Systemic Collaterals Locations**					
	**visits 0-1 used**			**all visits used**		
	**Estimate**	**95%CI**	***P* (for 0)**	**Estimate**	**95%CI**	***P* (for 0)**
for MET group at visit 0, with unstable Child-Pugh	0.0	0.0:0.3				
+ HCC group	1.4	0.9:1.8	<0.001			
+ stable Child-Pugh	0.2	-0.1:0.6	0.157			
+ additional visit						
If MET, any Child-Pugh	0.0	0.0:0.1	0.195	0.0	-0.1:0.1	0.913
If HCC, unstable Child-Pugh	-0.2	-0.1:0.6	0.155	0.2	0.1:0.4	0.008
If HCC, stable Child-Pugh	-0.1	-0.2:0.1	0.269	0.0	-0.2:0.2	0.976
+ ECOG	0.4	-0.1:0.9	0.084	–	–	–

Total Tumor Size at Baseline (normalized), Largest Tumor Size at Baseline (normalized), Number of Tumors at Baseline, TACE type, Microcatheter Location, Vascular Invasion, AFP and MELD dropped during model selection per AIC; dashed lines indicate that the corresponding variable was selected out by the model selection process as unimportant. Visit = TACE procedure.

Baseline total tumor size or largest tumor size and number of tumors, TACE type/selectivity, presence vascular invasion, and AFP/MELD values had no effect on odds of developing ascites.

**Figure 3:**
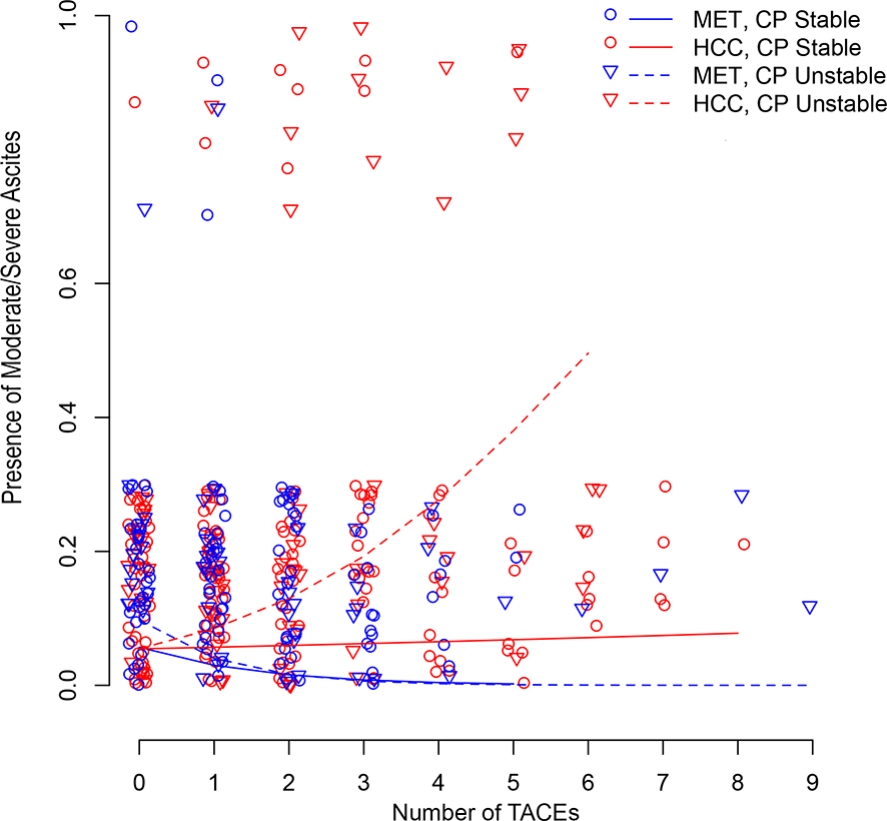
Scatter plot illustrating longitudinal changes over time in the development of moderate/severe ascites according to the number of TACEs and stratified by Child-Pugh (CP) stability (line = stable, dotted line = unstable) and metastatic (MET)/HCC groups. Each small shape (Δ or O) represents a patient’s presence at a TACE. Shapes are jittered for visualization purposes to avoid cluttering on top of each other. The linear time model in the log(Probability)/(1-log(Probability)) scale shows as a curve (nonlinear) model in the Probability scale.

**Figure 3** illustrates longitudinal changes over time in the presence of moderate/severe ascites.

### Longitudinal Analysis of Portosystemic Collaterals Formation

Baseline average number of portosystemic collaterals was significantly increased by 1.4 (95%CI:0.9–1.8) for the HCC group (*P*<0.001) (**Table 3B**). No metastatic patient demonstrated baseline portosystemic collaterals. The Child-Pugh status was not predictive of the average number of portosystemic collaterals after the first TACE regardless of the group (0.2 (95%CI: -0.1–0.6);*P*=0.157). However, an increased ECOG performance status at baseline demonstrated a trend for an increased number of portosystemic collaterals after the first TACE (0.4 (95%CI:-0.1–0.9);*P*=0.084) (**Table 3B**). Over the full follow-up, no significant change in the number of portosystemic collaterals was observed in metastatic patients, regardless of their Child-Pugh status (*P*=0.913), and in HCC patients with stable Child-Pugh (*P*=0.976). However, HCC patients with unstable Child-Pugh demonstrated an increase over time in the number of the portosystemic collaterals (*P*=0.008) (**Table 3B**).

Baseline total tumor size or largest tumor size and number of tumors, TACE type/selectivity, presence vascular invasion, and AFP/MELD values did not have statistically significant effect in the number of portosystemic collaterals.

**Figure 4:**
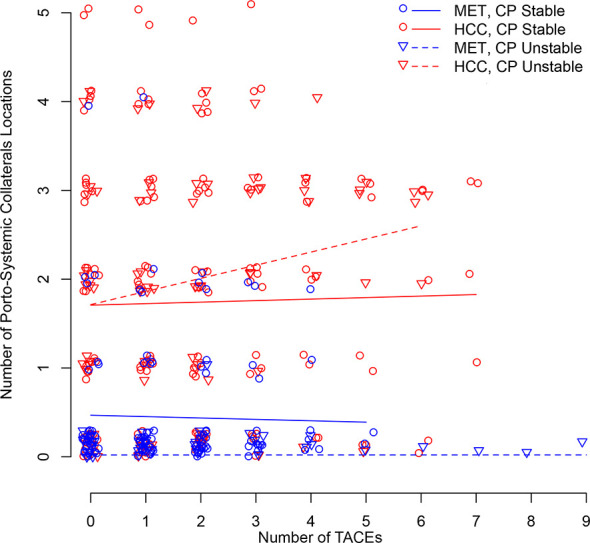
Scatter plot illustrating longitudinal changes over time in the number of portosystemic collaterals locations according to the number of TACEs and stratified by Child-Pugh (CP) stability (line = stable, dotted line = unstable) and metastatic (MET)/HCC groups. Each small shape (Δ or O) represents a patient’s presence at a TACE. Shapes are jittered for visualization purposes to avoid cluttering on top of each other. The linear time model in the log(Probability)/(1-log(Probability)) scale shows as a curve (nonlinear) model in the Probability scale.

**Figure 4** illustrates longitudinal changes over time in portosystemic collaterals.

### Portal Hypertension-Related Complications

Platelets transfusion was performed just before and during the first TACE in one HCC patient (1.75%) who underwent one procedure. No platelet transfusion after the initial TACE was required in the HCC group. Metastatic patients did not require platelet transfusions. Paracentesis was performed in one metastatic patient (2.4%) 7 weeks post-2^nd^TACE and in one HCC patient (1.75%) 9 weeks post-2^nd^TACE. No metastatic patient had variceal bleeding or encephalopathy. One HCC patient had variceal bleeding 4 weeks post-1^st^TACE. A month after variceal bleeding, the patient underwent a 2^nd^TACE followed by a 3^rd^TACE 8 weeks later, without subsequent complications. Another HCC patient had variceal bleeding complicated by encephalopathy and moderate ascites requiring paracentesis 8 weeks post-3^rd^TACE. Thus, early and delayed variceal bleeding (i.e. within 4 and 8 weeks of TACE, respectively) occurred in 0.4% of the procedures, respectively. Another HCC patient had a variceal bleed 5 months post-2^nd^TACE, and was likely unrelated to the procedure. All 3 patients with variceal bleeding underwent right lobar TACE. No correlation was noted between the number of TACEs and variceal bleeding risk. No patient developed hepato-renal/pulmonary syndromes.

## Discussion

It is well established that TACE is safe. However, scarce data exist on hemodynamic changes following TACE. In addition, the implications of repeated TACE on non-tumorous liver parenchyma, which is inevitably subjected to ischemic and drug-induced damage due to non-targeted delivery, remains largely unknown. As such, it is of clinical relevance to know whether TACE-related hemodynamic changes and liver damage translate into an increase in PVP, which may potentially lead to portal hypertension-related complications.

Circulatory changes in the portal/splanchnic venous system seem to appear immediately post-TACE and may remain elevated for some weeks (9, 12–14). However, these early modifications in PVBF measured on ultrasound do not necessarily translate in changes in HVPG (10, 11) and systematic increase in variceal pressure (12). These changes are part of a complex homeostatic process in which the embolization-related decrease in the hepatic arterial blood flow is compensated by a PVBF increase (12). The cause of these hemodynamic changes are not well established and are likely multifactorial. On top of arterial blood flow reduction consecutive to the embolization compensated by venous blow flow increment, other acute factors may be incriminated such as TACE-related production of cytokines and growth factors. This has to be integrated with the more complex and dynamic interactions of repeated therapy and its impact on liver parenchyma with architectural tissue changes over time (together with those related to chronic liver disease when present), and potential changes in liver function and cancer lesions.

Similarly to previous studies (9, 12–14), we investigated potential PVP changes after a single TACE. We demonstrated that the spleen volume significantly increased by 20cm^3^ after the first TACE (regardless of the group and Child-Pugh class change) with no significant impact on platelet count, ascites and number of portosystemic collaterals. Moreover, we examined whether the trend changes at different number of TACEs, including when only including up to two or more procedures, and we did not find any difference (data not shown).

The longitudinal analysis, including all TACEs, demonstrated that repeated TACE did not significantly influence platelet count over time in both groups, regardless of the liver function. This is an important finding as thrombocytopenia is a common issue in cancer patients, in particular in those with chronic liver disease (33). Although patients with more advanced chronic liver disease have a lower platelet count than those with compensated liver disease (34), our results highlight that even in HCC patients who experienced a worsening in their Child-Pugh class over the course of therapy, TACE does not impact platelet count. A significant increase in platelet count was observed in the metastatic group after the first TACE that could be attributed to an inflammatory reaction post-procedure. Importantly, no patient had to be newly transfused with platelets over the course of repeated TACEs.

By using a quantitative approach, we found that patients with a higher baseline tumor burden demonstrated a clear trend of having an increase in their spleen volume by 23cm^3^ after TACE (*P*=0.064). Moreover, the spleen volume tended to increase by 16cm^3^ with each round of TACE in both groups. Taken together these results highlight the fact that although repeated TACEs had an influence on PVP as seen by an increase in spleen volume, however these changes did not translate into a measurable and proportional hypersplenism as demonstrated by the absence of significant platelet count drop.

Both increased outflow resistance and portal venous inflow contribute to sinusoidal hypertension and results in formation of ascites (35). Thus, TACE may directly and indirectly contribute to the formation of ascites. We demonstrated that HCC patients in whom liver function remained stable throughout the course of repeated TACEs did not have an increased risk of developing clinically significant ascites. However, the odds of developing moderate/severe ascites significantly increased over time in HCC patients for whom the liver function decreased during rounds of TACE. Our results not only are in agreement with the well-established fact that progression of liver failure leads to the formation of ascites (4, 23, 35, 36), but also underscore that multiple TACEs *per se* do not lead to the formation of significant ascites in HCC patients with compensated liver disease. Interestingly, metastatic patients undergoing repeated TACEs had decreased odds of developing moderate/severe ascites, regardless of the Child-Pugh status. TACE-related decrease in tumor burden could be a potential explanation.

No significant change in the number of portosystemic collaterals was observed in metastatic patients, regardless of their liver function, and in HCC patients with stable Child-Pugh. Consistent with the natural history of cirrhosis, we found a clear link between decreased liver function and increase in PVP. Accordingly, HCC patients with unstable Child-Pugh demonstrated a statistically significant increase over time in the number of the portosystemic collaterals.

Despite the influence of TACEs on ascites as discussed above, only one (2.4%) and two (3.5%) patients in the metastatic and HCC groups, respectively, required paracentesis. TACE has been incriminated as a potential cause of variceal bleeding in patients with chronic liver disease, with early studies reporting a rate of 3-8% (37, 38). Our variceal bleeding rate (0.4%) is in agreement with recent series (0.3-1%) (39, 40) highlighting a better patient selection and improved technique.

The strengths of this work include the longitudinal study design, inclusion of HCC and metastatic patients, stratification of results according to liver function, and the use of sophisticated modeling approaches to evaluate interactions. Extensive subgroup analysis was done in both exploratory data analysis and post-model selection.

There were limitations. First, is the retrospective design. Second, our cohort is relatively small because we excluded many patients due to strict inclusion criteria aimed at decreasing missing data and increasing the validity of our results. Importantly, a power analysis determined the sample size was sufficient to detect with high probability the substantial treatment effects we had aimed to see in the study (>94%). Third, the study lacked HVPG measurements. However, we used validated surrogate markers of portal hypertension. Moreover, it is medically unjustifiable to obtain HVPG before and after each TACE. Forth, patients having a spleen too large to fit in the FOV of the MRI dataset were excluded, preventing the inclusion of the biggest spleen volume in the analysis.

In conclusion, repeated TACEs do seem to have an impact on PVP as evidenced by longitudinal analysis of non-invasive surrogate markers of portal hypertension. However, this increase in PVP has marginal effects, and with low portal hypertension-related complications.

## Data Availability Statement

The raw data supporting the conclusions of this article will be made available by the authors, without undue reservation.

## Ethics Statement

The studies involving human participants were reviewed and approved by Johns Hopkins Medicine IRB00231486. Written informed consent for participation was not required for this study in accordance with the national legislation and the institutional requirements.

## Author Contributions

CF: Design of the work, analysis, and interpretation of data, statistical analyses. JS: acquisition, analysis, and interpretation of data, statistical analyses. AB: acquisition and interpretation of data, revision of the work. JC, RS, JH, TP, and KH: acquisition and interpretation of data, revision of the work. ML: revision of the work, software technical assistance. RD: conception/design of the work, acquisition, analysis, and interpretation of data, drafting and revision of the work. All authors contributed to the article and approved the submitted version.

## Funding

Support for this work was provided by NIH/NCI R01 CA160771, P30 CA006973, Philips Research North America, Cambridge, MA, USA.

## Conflict of Interest

RD: Consultant: Boston Scientific/BTG, Guerbet. Grant Support: Boston Scientific/BTG, Guerbet, Society of Interventional Oncology. JC: Grant Support: Boston Scientific/BTG, Guerbet, Society of Interventional Oncology, Philips, Rolf W Guenther Foundation for Radiological Research. RS: grant support from the Max Kade Foundation and Siemens Healthineers. ML: former employee of Philips Research North America and is now an employee and stockholder of Visage Imaging, Inc. KKH: Advisory board for Boston Scientific, AStraZaeneca; Reasearch Support: Boston Scientific/BTG.

The remaining authors declare that the research was conducted in the absence of any commercial or financial relationships that could be construed as a potential conflict of interest.

## Publisher’s Note

All claims expressed in this article are solely those of the authors and do not necessarily represent those of their affiliated organizations, or those of the publisher, the editors and the reviewers. Any product that may be evaluated in this article, or claim that may be made by its manufacturer, is not guaranteed or endorsed by the publisher.

## Abbreviations

TACE, transarterial chemoembolization; PVP, portal venous pressure; HCC, hepatocellular carcinoma; PC, platelet count; HVPG, hepatic venous pressure gradient; PVBF, portal blood flow; ECOG. Eastern Cooperative Oncology Group performance status; MELD, Model for End-Stage Liver Disease; GEE, Generalized Estimating Equations; AIC, Akaike Information Criterion.
