# Supplementary material for: Longitudinal Analysis of the Effect of Repeated Transarterial Chemoembolization for Liver Cancer on Portal Venous Pressure
Source: Front Oncol. 2021 Nov 5;11:639235. doi: 10.3389/fonc.2021.639235 (PMC8602787; doi:10.3389/fonc.2021.639235)
Supplement: Supplementary file 1 [file DataSheet_1.docx]

Supplementary Material

# Supplementary material 1: TACE Protocol

All patients considered for TACE were discussed at our multidisciplinary liver tumor board. All TACE procedures were performed by experienced interventional radiologists by using a consistent approach. Briefly, the Seldinger technique was used to access the right common femoral artery and a 5-F vascular sheath was placed over a 0.035-inch Bentson guidewire (Cook, Bloomington, IN). A 5-F Simmons-1 catheter (Cordis, Miami Lakes, FL) was advanced under fluoroscopic guidance over the wire and reformed into the aortic arch, and used to select the celiac trunk. A 2.8-F Renegade HI-FLO microcatheter was then advanced over a Fathom-16 wire (Boston Scientific, Natick, MA) into the desired hepatic artery branch depending on the tumor location. Selective catheterization was performed to achieve lobar or sub-/segmental embolization based on the targeted lesions location. When cTACE was performed, an emulsion containing 50 mg of doxorubicin and 10 mg of mitomycin C in a 1:1 ratio with iodized oil (Lipiodol, Laboratoire Guerbet, Aulnay-sous-Bois, France) was infused and followed by of 100-300 μm microspheres (Embospheres, Merit Medical Systems, South Jordan, UT). When DEB-TACE was performed, a maximum of 100 mg doxorubicin per procedure loaded onto 100-300 µm microspheres was administered (DC Bead, Biocompatibles/BTG, Farnham, United Kingdom). Substantial arterial flow reduction to the tumor was defined as the technical endpoint of embolization. Complete occlusion of the tumor-feeding blood vessels was avoided to maintain the arterial pathway for potential re-treatment.

# Supplementary material 2: MRI Protocol

## MR imaging was performed at baseline and 3-6 weeks after each TACE by using a 1.5-T MRI unit (CV/I; GE Medical Systems, Milwaukee, WI) and a phased-array torso coil for signal reception. The standardized liver protocol included axial breath-hold unenhanced and contrast-enhanced (0.1 mmol per kilogram of body weight of intravenous gadodiamide [Omniscan, GE Healthcare, Princeton, NJ]) T1-weighted three-dimensional fat-suppressed spoiled gradient-recalled echo images (5.1/1.2; field of view, 320–400 mm2; matrix size, 192 × 160; section thickness, 4–6 mm; receiver bandwidth, 64 kHz; flip angle, 15°) in the arterial, portal venous and equilibrium phases (20, 60 and 180 seconds after intravenous contrast material injection, respectively).
